# Gas stripping assisted vapour permeation using graphene membrane on silicon carbide for ethanol recovery

**DOI:** 10.1038/s41598-023-37080-6

**Published:** 2023-06-16

**Authors:** Juan A. G. Carrio, VSSL Prasad Talluri, Swamy T. Toolahalli, Sergio G. Echeverrigaray, A. H. Castro Neto

**Affiliations:** Centre for Advanced 2D Materials, National University of Singapore, Singapore, 117546 Singapore

**Keywords:** Energy science and technology, Materials science, Nanoscience and technology

## Abstract

The conventional methods for ethanol recovery in low concentrations from diluted aqueous solutions are limited by the high energy consumed. Therefore, developing a cost-effective advanced membrane process for ethanol recovery and concentration is still necessary. A gas stripping-assisted vapour permeation (GSVP) process was applied to concentrate ethanol by the selective removal of water using hydrophilic graphene oxide (GO) membranes. Silicon carbide porous tubes were internally coated with GO-based membranes with an average thickness of 1.1 μm as a selective layer. Dry N_2_ was bubbled into the feed solution, carrying the saturated vapours to the separation module. The modified GSVP process was implemented to recover ethanol at lower temperatures than direct distillation and close-ended GSVP processes. The performance of the membrane-coated tubes was evaluated as a function of temperature and feed concentration, ranging from 23 to 60 °C and 10 wt% to 50 wt%. Distillates with 67 wt% and 87 wt% were obtained from feeds with 10 and 50 wt% ethanol at 50 °C, respectively. The evaporation energy spent by the modified GSVP process using GO-coated SiC tubes was 22% and 31% lower than the traditional distillation and vapour stripping processes.

## Introduction

The continuous depletion of fossil fuels and its impact on carbon footprint, together with the drastic increase in energy demand over its supplies, has created a significant void which needs to be addressed. Alternative energy resources, including biofuels, are being introduced to fulfil the present and future directions in a sustainable and environmentally friendly way. Among the most used biofuels is bioethanol, produced from biomass fermentation of feedstocks such as derivatives of edible plants and lignocellulosic biomass or, lately, by photosynthetic microbes and genetically altered photosynthetic microbes^1,2^. According to the US Department of Energy reports, global ethanol production increased from 13 billion gallons in 2007 to 29.03 billion in 2019. The production fell worldwide to 26.06 billion gallons in 2020 due to the COVID-19 pandemic^3^.

In recent years, the pervaporation (PV) process has been considered one of the alternatives to recover ethanol from its aqueous solution with relatively high flux and separation factor with low capital cost and energy consumption as per economic analysis reports^4^. The most common commercially available hydrophobic pervaporation membranes are made of polydimethylsiloxane (PDMS). They can be used to recover organics, and their advantages consist of easy production, low material cost and effective hydrophobicity^5^. However, using membranes often requires a condensation system with heat recovery that is less efficient than distillation systems with integrated heat recovery^6^. Many studies on the separation of ethanol–water by PV refer to the binary liquid mixture; however, in the case of recovering ethanol from the fermentation broth, the performance of the membrane and its long-term duty is affected by membrane fouling due to the direct contact of fermentation broth, which contains sugars, salts, bacterium, and other vital by-products^7^. A diversity of works has explored the use of different types of membranes, such as inorganic membranes, mixed matrix membranes and some functional membranes^5,8^. Fouling is practically unavoidable when the liquid comes directly in contact with the selective membrane. Hence, pre-treatment like microfiltration is required to avoid membrane damage before performing the PV process, which becomes inviable for practical applications^9,10^.

A separation technique that combines gas stripping with vapour permeation was introduced in the last decade to overcome the PV disadvantages of direct contact of fermentation broth with the membranes. Using a PDMS membrane, the gas stripping-assisted vapour permeation (GSVP) process was employed to recover furfural (2-furaldehyde) from lignocellulosic biomass. Compared to other techniques, the GSVP process required only 20% of the energy spent in distillation and around 44% less energy than PV^10^. In another study, the GSVP process was used to concentrate ethanol from its aqueous solution using a commercial PDMS membrane. A comparison with the PV process showed 10% increase in ethanol flux and 4.9 times in separation factor recovering ethanol from low-concentration solutions at 65 °C with long-time stability and without membrane fouling. The energy spent in evaporation was around 54% of the one required by distillation^9^.

Nanostructured graphene oxide (GO) membranes consist of stacked layers of oxidised graphene sheets separated by their oxygen-containing functional groups, forming a laminated structure of nanochannels. GO laminates have excellent characteristics that enable their application in membranes, like water dispersibility, hydrophilicity, negatively charged surface and production scalability. Therefore, GO-based membranes have been widely studied for their potential applications in water purification, organic solvent filtration, gas separation, and desalination via membrane distillation, among others^11–17^. In general, graphene-based membranes became an essential part of membrane distillation research^18^. This work implemented a modified GSVP process to recover and concentrate ethanol from feed solutions with low ethanol concentration. The modified process operates at lower temperatures than direct distillation and close-ended GSVP processes. A hydrophilic GO-based membrane was coated on the internal wall of a porous silicon carbide (SiC) ceramic tube as a selective layer to remove water from the vapour mainstream flowing through the tube.

## Results

### Membrane characterisation

**Figure 1:**
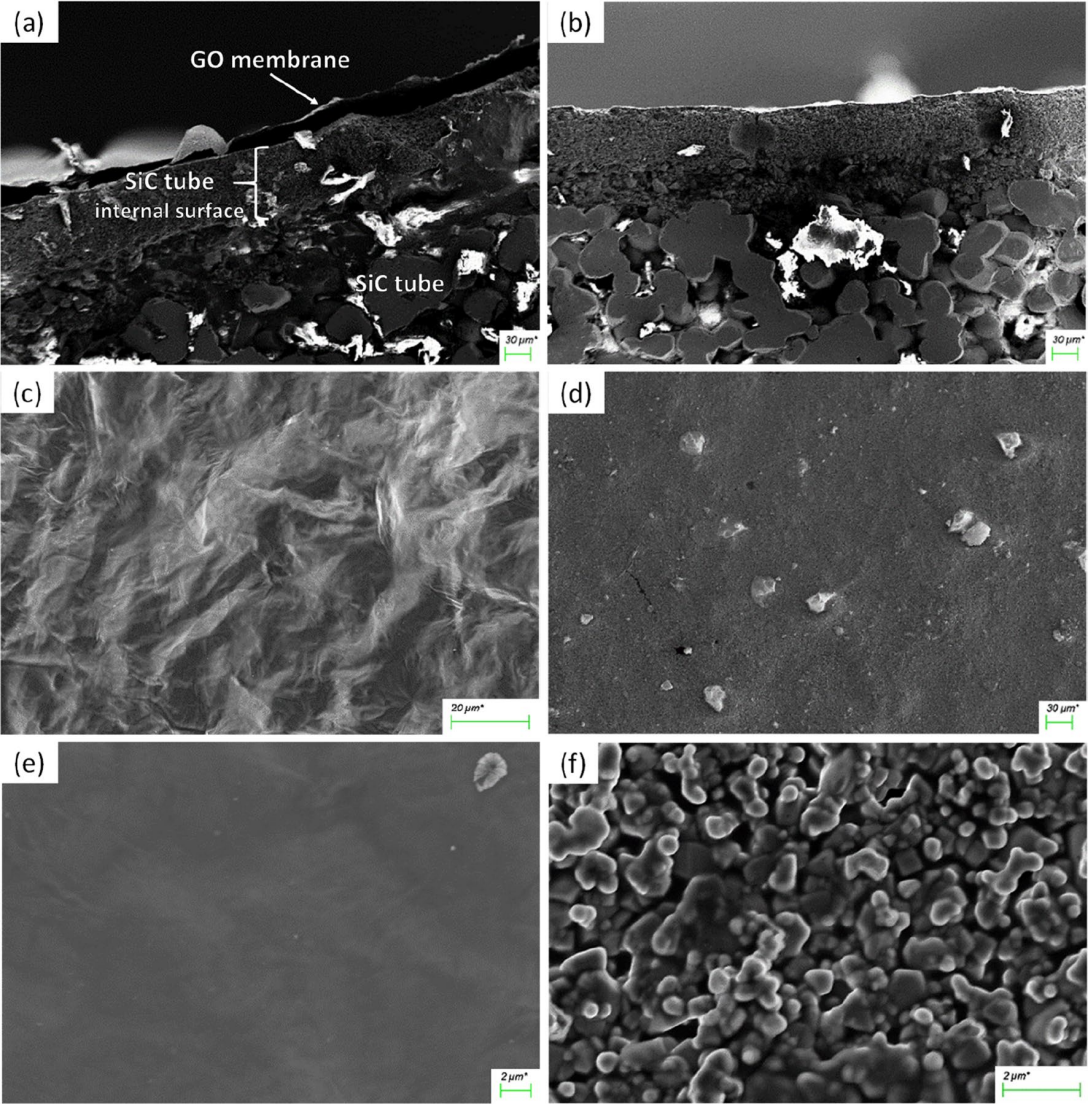
Fractured cross-section of (**a**) SiC/GO and (**b**) uncoated SiC tubes. The surface morphology of the GO membrane is shown in (**c**) and (**e**), and for comparison, the surface of uncoated SiC is shown in (**d**) and (**f**).

**Figure 2:**
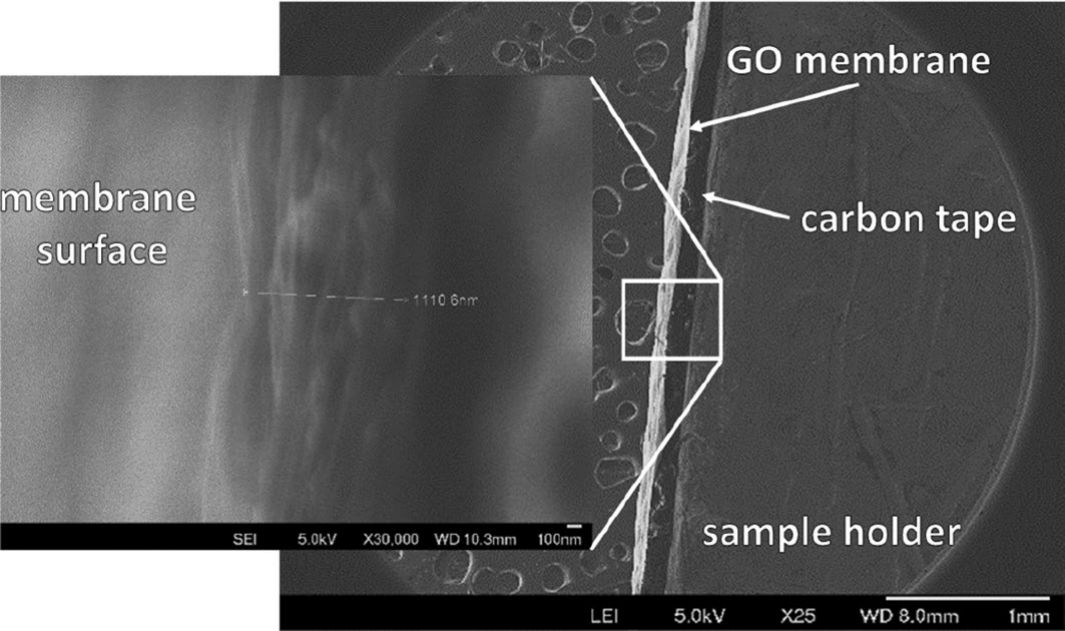
Cross-sectional micrographs of GO membrane—overview (right, scale bar of 1 mm) and thickness of 1110.6 nm (left, scale bar of 100 nm) of GO laminates.

The cross-section and surface morphology of the GO-coated tubes (SiC/GO) were analyzed by field emission scanning electron microscopy and compared to the uncoated original SiC tube. Figure 1a presents the cross-section of the SiC/GO sample, and Fig. 1b the cross-section of the uncoated SiC tube with the same magnification. The GO membrane on top of the ceramic surface is partially detached on the border due to the fracture applied to prepare the sample. The inner surface morphologies of the SiC/GO and SiC tube are compared side by side in Fig. 1c–f. The GO membrane's smooth, continuous, and undulated surface covers the porous surface of the SiC, which is shown uncoated in Fig. 1d–f. The pore size of around 600 nm of SiC can be observed in Fig. 1d. A sample of GO membrane was detached from the SiC and fixed to the sample holder by carbon tape, shown in the cross-sectional overview Fig. 2. Highlighted, the measurement of its regular thickness of 1.1 μm and the stacked microstructure of GO is also presented.

**Figure 3:**
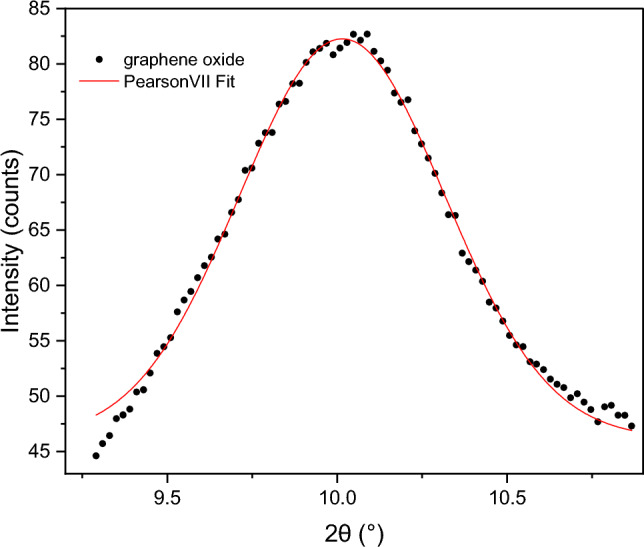
XRD Bragg reflection of (100) planes of a sample GO membrane.

A sample membrane was detached from the SiC tube to collect X-ray diffraction (XRD) data. The diffractometer was set in step-scan mode with steps of 0.02° with a counting time of 1 s. The peak at 2θ = 10.01° in Fig. 3 corresponds to the Bragg reflection of the planes (100) of GO, with an interplane distance of 8.82 Å (0.882 nm), calculated using the Bragg equation and the angular position from a peak fitting with the function Pearson VII.

### GSVP results

#### Effect of the temperature on the GSVP process

**Figure 4:**
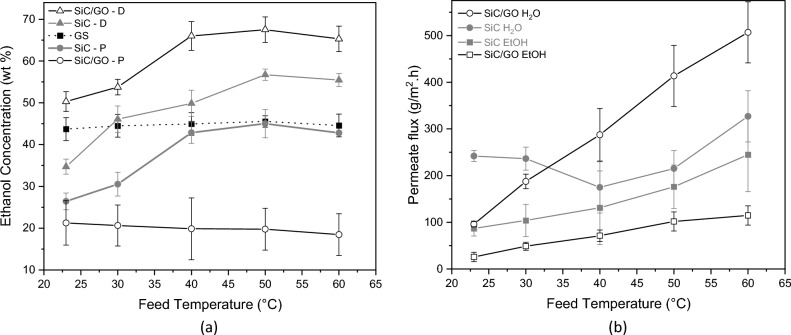
(**a**) Ethanol concentration in distillate and permeate as a function of the temperature using a feed concentration of 10 wt% ethanol. (**b**) Permeate fluxes of ethanol and water in SiC and SiC/GO as a function of the temperature for a feed concentration of 10 wt% ethanol.

The condensation of the collected vapours from gas stripping (GS) is a distillate with a constant concentration of around 44 wt% ethanol over the whole temperature interval (Fig. 4a). On the other hand, the results of GSVP consist of two streams, namely distillate (D) and permeate (P), that show differences for SiC and SiC/GO. For pure SiC, the condensation of vapours inside the porous ceramic walls favours the circulation of ethanol in the mainstream at temperatures higher than 30 °C due to its higher vapour pressure, so the distillate is richer in ethanol than in the case of GS, whilst the permeate tends to have the same concentrations of GS. A significant difference between the ethanol-rich distillate and the water-rich permeate of SiC/GO can be observed in the whole temperature interval compared to the distillate and the permeate of pure SiC. Figure 4a shows the effect of the GO membrane in the SiC porous tube, demonstrating the selective extraction of water vapour and its transport to the permeate side of the tube. At 50 °C, the distillate of SiC/GO reaches its maximum value of 67.5 wt%, while its permeate remains around 20 wt% ethanol for all evaluated temperatures.

The fluxes of ethanol and water through SiC and SiC/GO walls to the permeate side are plotted as a function of the temperature in Fig. 4b. For temperatures > 30 °C, the flux of water through the GO membrane is higher than through SiC, and correspondingly, the ethanol flux is lower. Below 30 °C, water vapour is transported at a higher speed inside the pores of pure SiC because of the low ethanol vapour pressure and its low concentration. In these conditions, the SiC pores of 600 nm size can transport water and ethanol vapour to the permeate side at a higher speed than the GO nanochannels of around ~ 1 nm size. The GO nanochannels have a size of approximately 1 nm, based on the observed interlayer distance by XRD (~ 0.8 nm) and considering the presence of nano slits between the GO flakes and flake defects in the form of nanopores.

**Figure 5:**
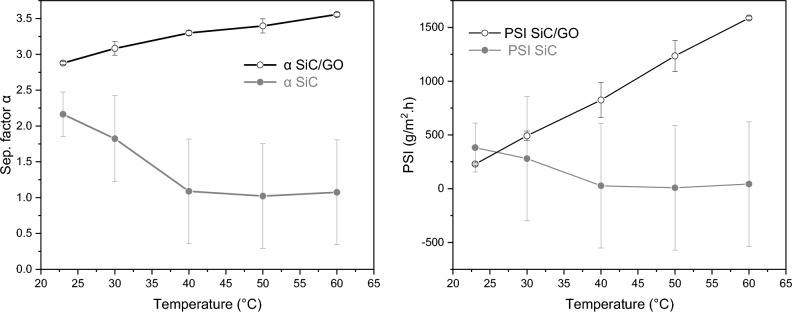
Permeate α (left) and PSI (right) as a function of the temperature with feed concentration of 10 wt% ethanol.

The results show (Fig. 5) an evident selectivity of water over ethanol for the system SiC/GO, characterised by its higher values of the separation factor α and Process Separation Index (PSI) for temperatures > 30 °C.

#### Effect of feed concentration on the GSVP process

During the production of bioethanol by the fermentation process, the ethanol concentration in the fermentation broth varies and is influenced by raw materials, microorganisms, and the reactor operating parameters^2,19^. Hence, to investigate the performance of the GSVP process as a function of the feed concentration, a series of experiments were performed with the feed at concentrations of 10, 20, 30, 40 and 50 wt% at a fixed temperature of 50 °C. GS experiments were also performed in the same conditions as GSVP.

**Figure 6:**
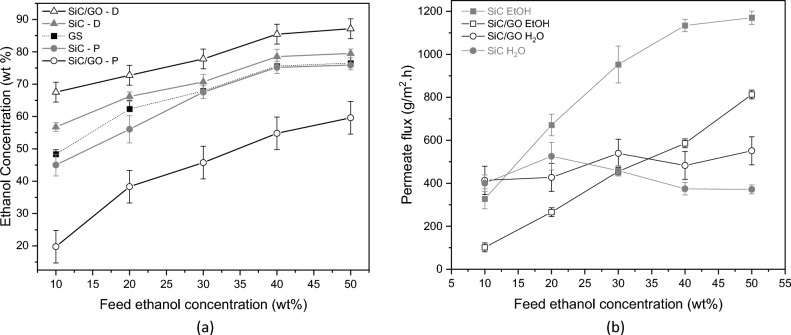
(**a**) Ethanol concentrations for GSVP and GS at 50 °C as a function of the feed concentration. (**b**) Permeate fluxes of ethanol and water as a function of the feed concentration at 50 °C for SiC and SiC/GO.

The distillate and permeate obtained for SiC (Fig. 6a) show similar behaviour and values to the GS distillate, indicating SiC tube is not selective. In contrast, the values for SiC/GO are clearly apart from GS, showing the water selectivity effect of GO membrane. The highest ethanol concentrations of distillate were obtained for the SiC/GO system; correspondingly, its permeate had the lowest concentrations. As an example of the performance, the ethanol concentration was increased by 48 wt% on average. Feed concentrations of 10 and 50 wt% resulted in distillates of 67 and 87 wt% ethanol, respectively.

The ethanol vapour pressure increases with the feed concentration, so it passes more easily through the pores of the tube. This effect can be observed by the permeate flux of ethanol, shown in Fig. 6b, which increases almost linearly with the feed concentration for SiC/GO and until 40 wt% for SiC. For SiC/GO, the flux of ethanol is substantially lower than those of SiC in the whole interval of concentrations evaluated due to the resistance imposed by the GO membrane, only surpassing the water flux for concentrations > 30 wt%.

**Figure 7:**
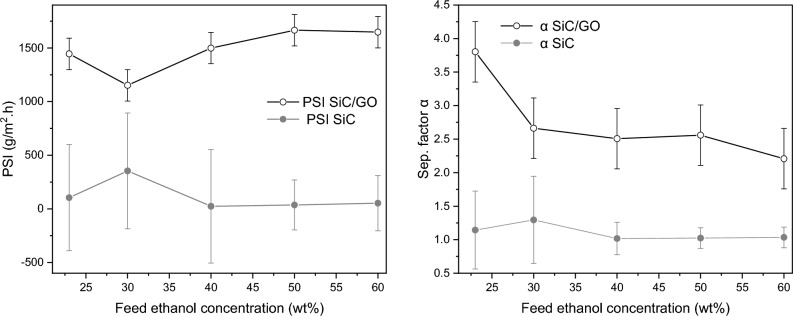
Permeate separation factor (α) and PSI as a function of the feed concentration at 50 °C for SiC and SiC/GO.

The permeate water flux of SiC/GO, even under the high vapour pressure of ethanol, remains higher than that of SiC for feed concentrations above 20 wt% (Fig. 6b). The permeate separation factor and the process separation index for the interval of feed concentrations are plotted in Fig. 7, where a remarkable selectivity to water can be observed for SiC/GO.

#### Energy calculation and economic viability

**Figure 8:**
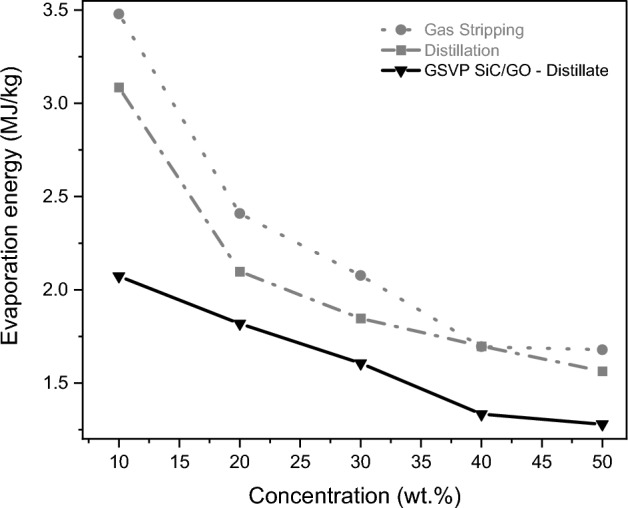
Evaporation energy for the GSVP, GS and distillation processes at 50 °C as a function of the concentration.

The most crucial parameter for economic considerations is energy consumption, which corresponds mainly to evaporation energy. It was calculated using Eq. (6) for the processes GS, GSVP with SiC/GO, and pure distillation using data obtained from the vapour-liquid equilibrium of ethanol/water at 50 °C. Comparing the evaporation energies, GSVP with SiC/GO is the less energy-spending process for all considered feed concentrations, as seen in Fig. 8. On average, GSVP with SiC/GO consumes around 20% less energy than distillation processes, reaching 33% for a feed concentration of 10 wt%. Regarding materials, around 0.25 mg/cm^2^ of GO on SiC tubes were used for producing the membranes. Considering a high-quality GO price of $10,000 USD/kg, the membrane cost would be about $0.0025 USD/cm^2^.

## Discussion

The size of the nanochannels and slits between flakes and the interaction of water with the functional groups on GO laminates-based membranes can explain the selective effect of water detected, as described in detail in the literature^16,20,21^. When soaked in pure water, the interlayer space remains around 0.8 nm, as initially measured by XRD in dry conditions. With time, the membrane hydrates and the interlayer space increases up to ~ 7 nm^19^. The interlayer distance when soaking in ethanol/water mixtures is smaller than in pure water^17^. In the GSVP process, the GO membrane is exposed to a mixture of water and ethanol vapours, which can be considered a dried/semi-dried condition, with the interlayer spacing increasing only up to about 1.2 nm^16^.

**Figure 9:**
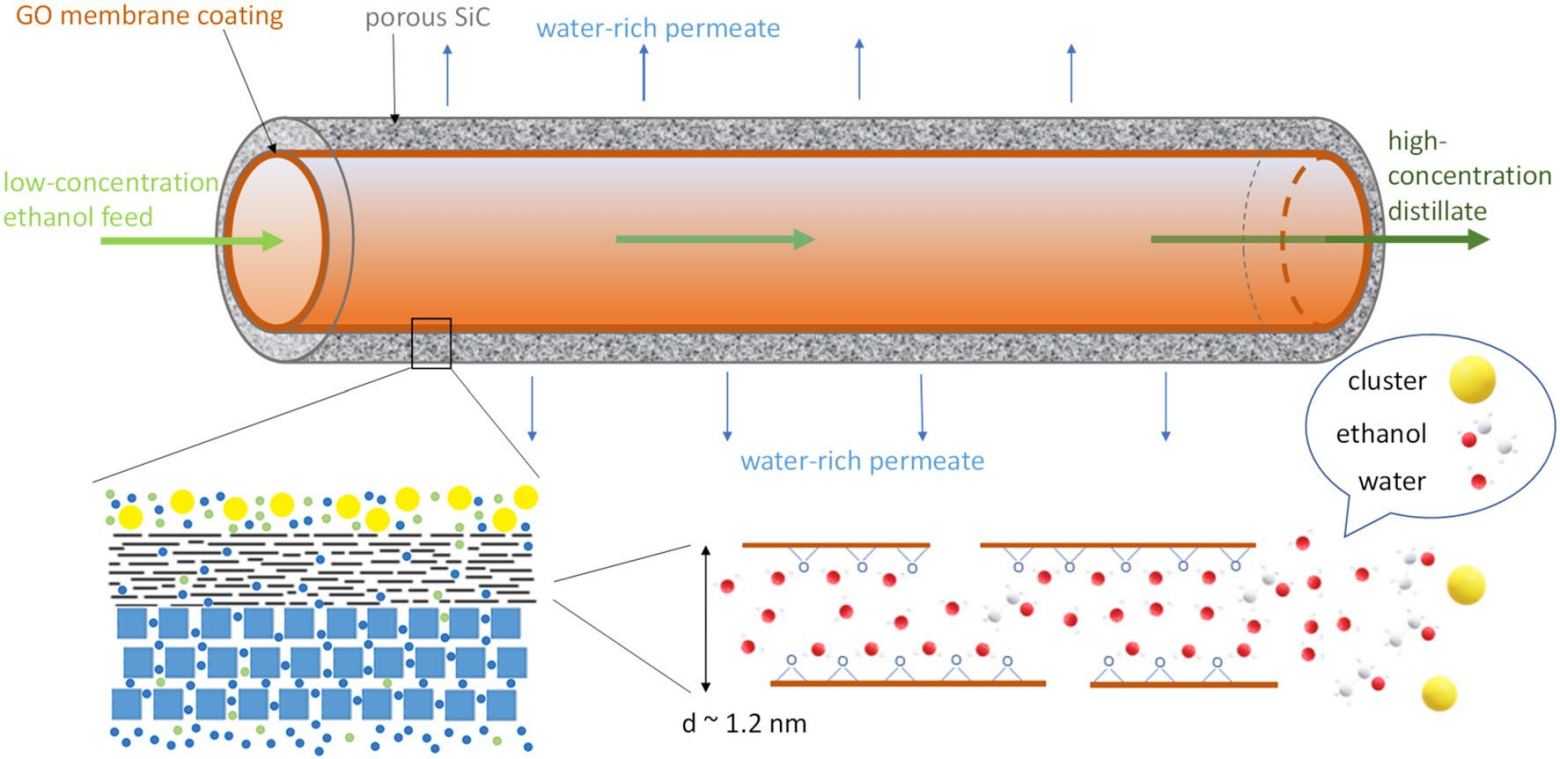
Schematic representation of the separation process inside the nanochannels in the membrane coated on porous SiC. The blue and green dots represent the water and ethanol molecules, respectively.

The oxygen-containing functional groups of GO are responsible for the mobility of water molecules through the laminated membranes in the form of a semi-ordered water network with 30% higher density than pure water. These liquid-like layers, represented in the inset of Fig. 9, are rapidly inserted and de-inserted in the GO membrane structure. They are believed to be responsible for fast and efficient water permeation^17^. Consequently, the water network strongly hinders the mobility and passage of water/ethanol clusters and ethanol molecules, which are bigger in size than the water molecule. This effect explains the larger differences in the permeate fluxes of water to ethanol for the SiC/GO membrane than for the SiC membrane, as shown in Fig. 4b. Additionally, the higher flux of permeate water than ethanol for low feed concentrations is observed in SiC/GO, as shown in Fig. 6b. In contrast, for SiC, the flux of ethanol in the permeate tends to be higher than that of water from approximately 15 wt% feed concentration (Fig. 6b).

Summarizing, the GSVP process was modified using a novel design with GO-coated tubes that extract water perpendicularly from the mainstream ethanol/water mixture vapours. Our system consists of porous SiC tubes with an internal and continuous hydrophilic graphene oxide coating, which allows the selective diffusion of water and its transport to the permeate side. Dry N_2_ gas was bubbled into the feed solution to carry saturated vapours into the separation module, avoiding the introduction of moisture and increasing the overall safety due to its inert nature. The process operation was studied for feed concentrations ranging from 10 to 50 wt% ethanol and temperatures from 23 to 60 °C. The temperature range evaluated is substantially lower than typical temperatures applied for ethanol separation. Using the GO membrane increased ethanol's flux and concentration downstream, optimising the process output. The system's operational lifetime was observed to be at least 3 months due to no observable membrane fouling from exposition to water and ethanol. A gradual delamination of the GO membrane from SiC was observed when heating the system over to 60 °C. Therefore, to ensure longer-term tests, it is recommended to use a working temperature of around 50–55 °C.

Inside the SiC/GO tube, the stream composition changed from water-rich to ethanol-rich due to the selective diffusion of water vapour through the inner hydrophilic GO-coated wall. The best results in terms of ethanol concentration in the distillate were found for temperatures > 30 °C. Using feeds containing 10 wt% and 50 wt% ethanol at 50 °C, the distillates obtained contain 67 wt% and 87 wt% ethanol, respectively. Regarding energy, our modified GSVP process consumes less than the traditional distillation process independent of the feed concentration, reaching up to 33% less for the lower-feed concentration evaluated.

Although the separation process was designed to selectively remove the water content from an ethanol–water vapour mainstream flowing through the tubes, its application can be extended to recovering volatiles from fermentation processes via integrating with a bioreactor. Furthermore, this system can also be adapted for water desalination by membrane distillation, considered the most important among the many membrane distillation applications.

## Methods

### Materials

Ethanol/water mixtures were prepared by wt/wt ratios (Absolute Ethanol 99.8%, Fisher Scientific International Inc., UK). The ethanol concentration in mixtures was assessed with a density meter (DMA 4500 M, Anton Paar, Austria). A diaphragm pump (MVP 015–4, Pfeiffer, Germany) established the vacuum pressure. A jacketed round bottom reactor was used as feed tank, and a heating circulator (CORIO CP BC12, Julabo GmbH, Germany) was used to control the jacket temperature. Commercial SiC single-channel tubes with internal Ø of 1.7 cm, external Ø of 2.5 cm, length of 21 cm, and 600 nm pore size (CRYSTAR®, Saint-Gobain Industrie Keramik Rödental GmbH, Germany) were pre-treatment in a hot air oven at 60 °C for 8 h to remove any trapped moisture and solvent impurities inside the pores. The graphene oxide paste (Graphene Oxide 10% water washed aqueous past, Abalonyx Innovative Materials, Norway) was dispersed and ultrasonicated for 3 h in deionised water to obtain a stable and uniform 1 mg/ml suspension.

### Membrane caracterization

A fracture was applied to an original SiC tube and a SiC/GO tube for Field Emission Scanning Electron Microscopy (FESEM) characterization. In addition, a sample of the GO membrane was detached from the SiC and fixed to the FESEM sample holder using carbon tape. The specimens were coated with a gold layer of approximately 5 nm thickness and analyzed using Carl Zeiss AG—SUPRA 40 equipment. X-ray diffraction data were collected in a standard laboratory diffractometer Rigaku-Dairix with CuKα radiation and Bragg–Brentano geometry.

### Deposition of GO membrane on SiC tubes

The pre-treated SiC porous tube was inserted and fixed in a homemade cylindrical membrane separation module. The module was set in vertical position and connected to the feed and permeation sides of the experimental setup, as illustrated in Fig. S1. The tube's inner part was connected to the feed module side, and the outer part was connected to the permeate module side.

The GO water suspension was pumped from the feed tank into the bottom inlet of the module. The peristaltic pump was set to circulate the suspension at a constant flow of 0.02 l/min for 30 min. The pressure on the permeate side was kept at 0.1 bar, and a condensation system collected the permeate liquid. The coating was finished by draining the suspension using the pump in reverse flow of 0.01 l/min. After extracting the suspension, N_2_ gas was blown through the tube's inner side for 30 min keeping the pressure on the permeate side at 0.1 bar. The module was disconnected from the circular system and dried with airflow for at least 24 h. Four coating rounds were applied to form a regular membrane with a thickness of approximately 1.1 μm and total membrane area of approximately 112 cm^2^. Before each round, the module was rotated 180° for the liquid to be pumped into the other end of the tube. The total consumption of GO was about 0.25 mg/cm^2^ of the tube's inner wall.

### Gas stripping-assisted vapour permeation with membrane separation

The GSVP experiments were conducted by fixing the sample tube in the module mentioned above. The tube's inlet and outlet were connected to the feed tank and to the distillate collection system, respectively. The external wall was connected to the permeate collection system, as shown in the process flow diagram of Fig. S2. A carrier gas (N_2_) was bubbled into the feed solution at a flow rate of 1.0 l/min to enhance the formation of saturated vapours. A mainstream of saturated vapours and N_2_ was driven by pressure difference from the feed tank to the membrane module alongside the sample tube, from which it was directed to a cold trap for condensation and collection as distillate at 1.0 bar pressure. The permeate passes through the tube wall driven by the low pressure of 0.1 bar established in the permeate side of the tube. The permeate was collected by a cold trap connected to a vacuum pump.

To study the effect of temperature on the GSVP process, experiments were conducted with feed-solution temperatures of 23, 30, 40, 50, and 60 °C and a fixed feed concentration of 10 wt%. The condensates were weighted every 90 min. All vapour flow lines were thermally insulated to minimise heat losses (Fig. S3). All experiments were initiated after 30 min to ensure membrane saturation and were performed at a steady state. Standard deviations were calculated from the data obtained from at least five experimental repetitions. For comparison, pure GS experiments were performed using the same setup and under the same conditions without the porous tube.

### Analytical method

The membrane separation performance is, in general, evaluated by the parameters flux (J) and separation factor (α), which are defined by Eqs. (1) and (2), respectively:

1 $$ J = \frac{m}{A \times \Delta t} $$

2 $$ \propto = \frac{{y_{i} /\left( {1 - y_{i} } \right)}}{{x_{i} /\left( {1 - x_{i} } \right)}} $$

where m is the mass transported through a membrane of effective area A during the time interval ∆t, and x_i_ and y_i_ are the mass fraction of component *i* in the feed and permeate solution, respectively.

In the case of a combined separation of gas stripping (α_strip_) and vapour permeation through a membrane (α_mem_), the overall separation factor (α_GSVP_) must consider both processes, as per Eq. (3)

3 $$ \begin{aligned} & \propto_{GSVP}\, =\, \propto_{strip} \times \propto_{mem} \\ & \quad = \frac{{y_{strip,i} /\left( {1 - y_{strip,i} } \right)}}{{x_{i} /\left( {1 - x_{i} } \right)}} \times \frac{{y_{i} /\left( {1 - y_{i} } \right)}}{{y_{strip,i} /\left( {1 - y_{strip,i} } \right)}} \\ & \quad = \frac{{y_{i} /\left( {1 - y_{i} } \right)}}{{x_{i} /\left( {1 - x_{i} } \right)}} \\ \end{aligned} $$

where y_strip,i_ is the mass concentration of component *i* in the mixture of saturated vapours after stripping.

Hence the membrane separation factor α_mem_ can be obtained by the quotient

4 $$ \propto_{mem} = \frac{{ \propto_{GSVP} }}{{ \propto_{strip} }} $$

The performance of the process is also usually expressed through the process separation index PSI, which combines the separation factor and the flux. Here the GSVP process separation index of component *i* is defined by

5 $$ PSI_{i} = \pm J_{i} \times \left( { \propto_{GSVP} - 1} \right) $$

Finally, the evaporation energy for the processes at a due temperature can be calculated using the evaporation energy of the components and the separation factor by Eq. (6)

6 $$ Q_{norm}^{ev} = H_{EtOH}^{ev} + H_{{H_{2} O}}^{ev} \times \left( {\frac{{1 - x_{EtOH} }}{{ \propto_{{EtOH/H_{2} O}} \times x_{EtOH} }}} \right) $$

where *H* represent the evaporation heat of ethanol and water, *x*_*EtOH*_ is the ethanol concentration in the feed solution, and α_EtOH/H2O_ is the overall separation factor in the processes^9^.

## Supplementary Information

Supplementary Information.

## Data Availability

The datasets generated during and/or analysed during the current study are available from the corresponding author on reasonable request.
