# Supplementary material for: Gas stripping assisted vapour permeation using graphene membrane on silicon carbide for ethanol recovery
Source: Sci Rep. 2023 Jun 16;13:9781. doi: 10.1038/s41598-023-37080-6 (PMC10275899; doi:10.1038/s41598-023-37080-6)
Supplement: Supplementary file 1 — Supplementary Information. [file 41598_2023_37080_MOESM1_ESM.docx]

**Supplementary Information to Gas stripping assisted vapour permeation using graphene membrane on silicon carbide for ethanol recovery**

Juan A. G. Carrio^1,^, VSSL Prasad Talluri^1^, Swamy T. Toolahalli^1^, Sergio G. Echeverrigaray^1^, A. H. Castro Neto^1^

^1^Centre for Advanced 2D Materials, National University of Singapore, 117546, Singapore

^*^juan.carrio@nus.edu.sg

Table S1: The GSVP of 10wt% ethanol/water mixture by SiC/GO membrane at different temperatures

| **Temperature (^o^C)** | **Ethanol concentration in Vapour phases (wt.%)** | | | **Flux (g/m^2^.h)** | | **Separation factor and Separation factor index of Ethanol phase** | | | | **Separation factor and Separation factor index of water phase** | | | |
| --- | --- | --- | --- | --- | --- | --- | --- | --- | --- | --- | --- | --- | --- |
|  | **Gas Stripping** | **Permeate** | **Distillate** | **Permeate** | **Distillate** | **Overall SP** | **L-V phase transition SP** | **membrane SP** | **SP index (PSI)** | **Overall SP** | **L-V phase transition SP** | **membrane SP** | **SP index (PSI)** |
| 23 | 43.7 | 21.3 | 50.3 | 121.9 | 154.5 | 2.42 | c | 0.4 | 173.2 | 0.4 | 0.1 | 2.9 | 56.3 |
| 30 | 44.5 | 20.6 | 53.8 | 236.4 | 290.2 | 2.33 | 7.2 | 0.3 | 314.7 | 0.4 | 0.1 | 3.1 | 107.1 |
| 40 | 45.0 | 19.9 | 66.0 | 358.7 | 423.7 | 2.22 | 7.3 | 0.3 | 438.2 | 0.5 | 0.1 | 3.3 | 158.1 |
| 50 | 45.6 | 19.8 | 67.5 | 515.4 | 715.1 | 2.21 | 7.5 | 0.3 | 623.1 | 0.5 | 0.1 | 3.4 | 226.3 |
| 60 | 44.6 | 18.5 | 65.3 | 621.4 | 1168.8 | 2.03 | 7.2 | 0.3 | 639.7 | 0.5 | 0.1 | 3.6 | 257.1 |

Table 2: The GSVP of ethanol/water mixture by SiC/GO membrane at different ethanol concentrations of feed solution

| **EtOH concentration. in feed solution (wt.%)** | **Ethanol concentration in Vapour phases (wt.%)** | | | **Flux (g/ m^2^.h)** | | **Separation factor and Separation factor index of Ethanol phase** | | | | | **Separation factor and Separation factor index of water phase** | | | |
| --- | --- | --- | --- | --- | --- | --- | --- | --- | --- | --- | --- | --- | --- | --- |
|  | **Gas Stripping** | **Permeate** | **Distillate** | **Permeate** | **Distillate** | **Overall SP** | **L-V phase transition SP** | **membrane SP** | **SP index (PSI)** | **Overall SP** | | **L-V phase transition SP** | **membrane SP** | **SP index (PSI)** |
| 10 | 48.4 | 19.8 | 67.5 | 515.4 | 715.1 | 2.21 | 8.40 | 0.26 | 623.09 | 0.45 | | 0.12 | 3.80 | 282.1 |
| 20 | 62.3 | 38.3 | 72.8 | 692.7 | 864.5 | 2.41 | 6.43 | 0.38 | 979.65 | 0.41 | | 0.16 | 2.66 | 405.8 |
| 30 | 67.9 | 45.8 | 77.8 | 994.4 | 1083.8 | 1.93 | 4.83 | 0.40 | 922.80 | 0.52 | | 0.21 | 2.51 | 478.6 |
| 40 | 75.6 | 54.8 | 85.5 | 1068.7 | 1122.4 | 1.82 | 4.65 | 0.39 | 874.78 | 0.55 | | 0.21 | 2.56 | 481.0 |
| 50 | 76.5 | 59.6 | 87.1 | 1364.1 | 1736.3 | 1.44 | 3.17 | 0.45 | 593.51 | 0.70 | | 0.32 | 2.21 | 413.6 |

Table 3: Evaporation energy comparisons between GSVP, gas stripping and distillation process at 50^o^C feed Temperature

| **Feed** | **GSVP Process (Distillate)** | | | **Gas stripping Process** | | | **Distillation Process** | | | **Evaporation Energy Comparison (%)** | | | |
| --- | --- | --- | --- | --- | --- | --- | --- | --- | --- | --- | --- | --- | --- |
| **EtOH (wt.%)** | **GSVP Distillate (Wt.%)** | **Distillate SP** | **Q EtOH (MJ/Kg)** | **Gas Stripping (wt.%)** | **L-V phase transition SP** | **Q EtOH (MJ/Kg)** | **VLE Distillate (wt.%)** | **Distillation SP** | **Q EtOH (MJ/Kg)** | **GSVP Vs GS (%)** | **GSVP Vs Distillation (%)** | **GS Vs Distillation (%)** |  |
| 10 | 67.5 | 18.7 | 2.1 | 48.4 | 8.4 | 3.5 | 52.5 | 9.9 | 3.1 | 50.6 | 39.3 | 12.0 |  |
| 20 | 72.8 | 10.7 | 1.8 | 62.3 | 6.4 | 2.4 | 67.0 | 8.1 | 2.1 | 27.9 | 14.3 | 13.8 |  |
| 30 | 77.8 | 8.2 | 1.6 | 67.9 | 4.8 | 2.1 | 72.2 | 6.0 | 1.9 | 25.6 | 13.9 | 11.7 |  |
| 40 | 85.5 | 8.8 | 1.3 | 75.6 | 4.7 | 1.7 | 75.6 | 4.6 | 1.7 | 23.9 | 24.1 | -0.2 |  |
| 50 | 87.1 | 6.8 | 1.3 | 76.5 | 3.2 | 1.7 | 78.9 | 3.7 | 1.6 | 27.1 | 20.0 | 7.1 |  |
| Average | | | | | | | | | | 31.0 | 22.3 | 8.9 | |

**Laboratory setup**

|  |
| --- |

Figure S1: Process flow diagram for (a) GO membrane coating in SiC porous tube and (b) membrane drying by N_2_ gas flow.


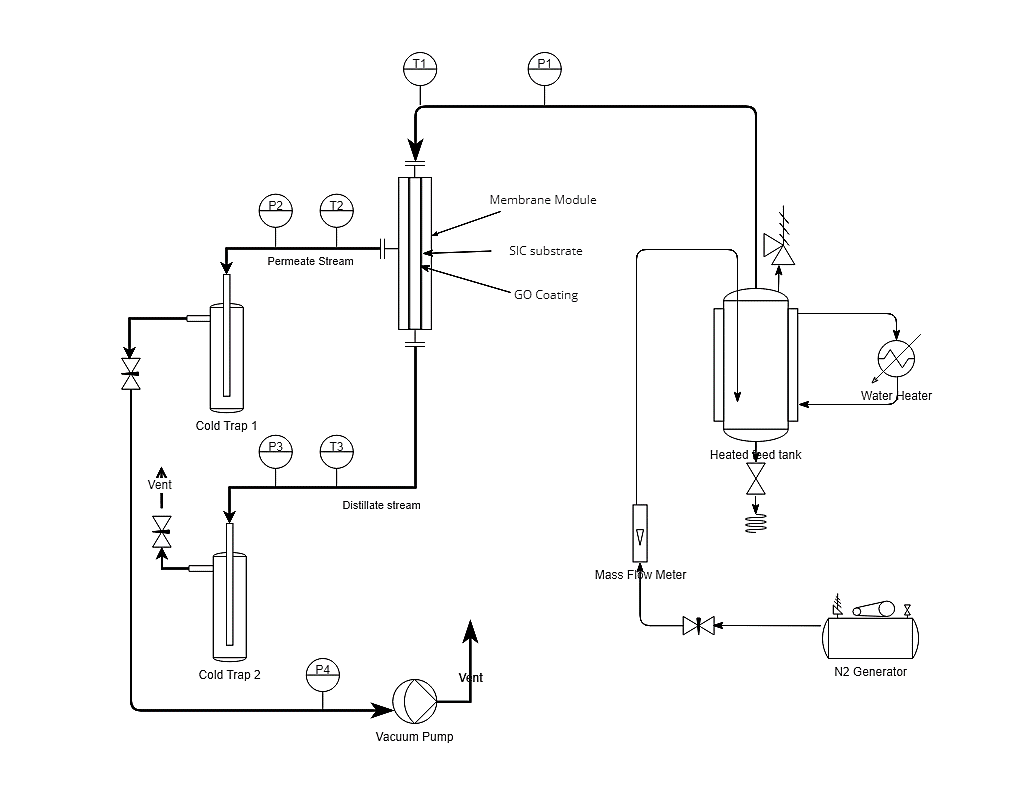


Figure S2: Process flow Diagram of GSVP experimental setup


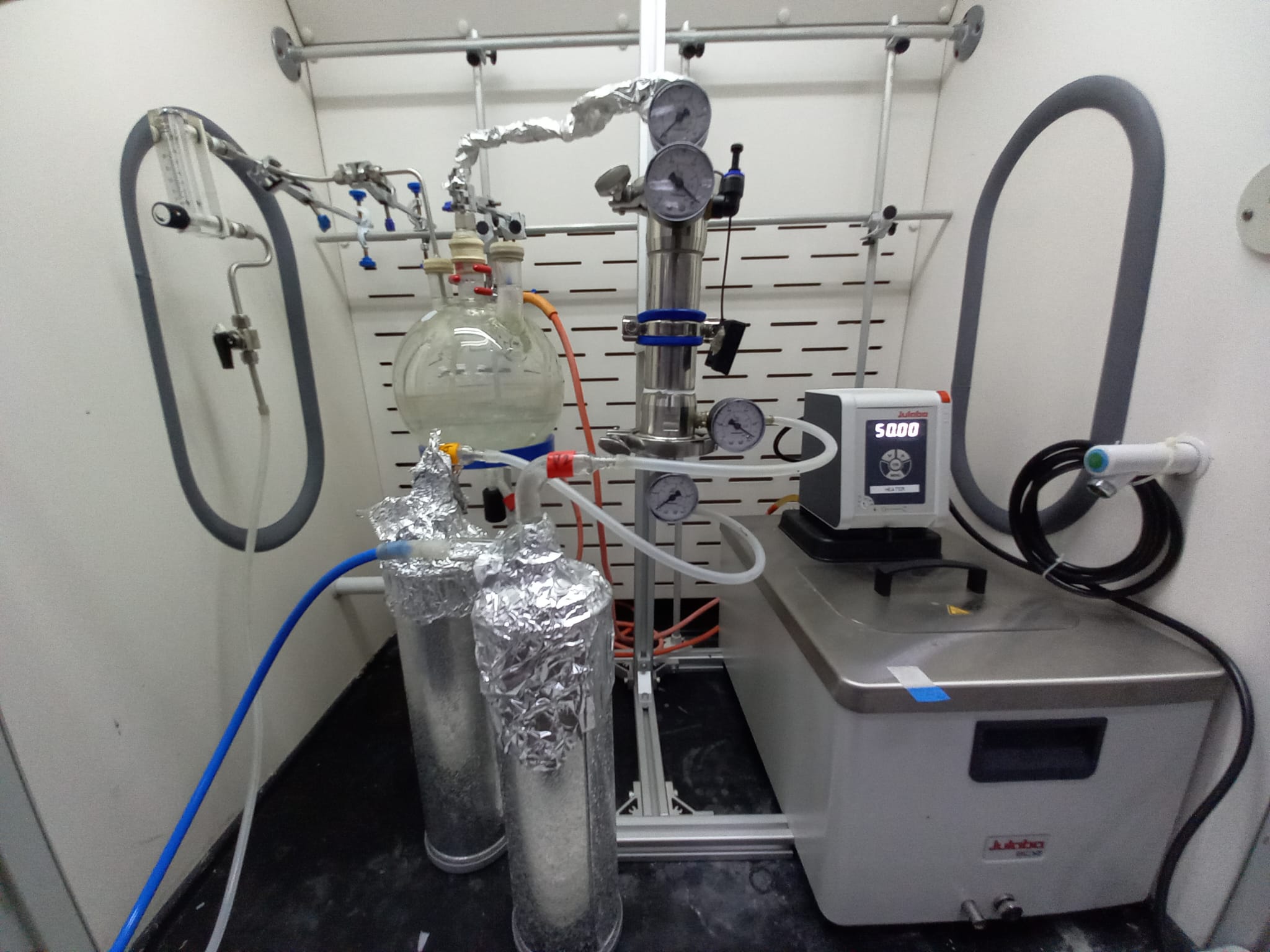


Figure S3: Laboratory setup.
